# Specialty Grand Challenge—Assisted Reproduction

**DOI:** 10.3389/frph.2021.551499

**Published:** 2021-07-26

**Authors:** Eitan Lunenfeld

**Affiliations:** Department of Obstetrics and Gynecology, Soroka Medical Center, Faculty of Health Sciences Ben Gurion University of the Negev, Beer Sheva, Israel

**Keywords:** coronavirus disease, artificial gametes, genetic engineering, non-invasive preimplantation genetic testing, embryonic stem cells, induced pluripotent stem cells

## Introduction

The relatively “modern” assisted reproductive technology (ART) started with the first successful induction of ovulation followed by a pregnancy with the use of menopausal urinary products in 1963 (1). At around the same time, the use of sperm that had been frozen with liquid nitrogen and thawed resulted in the first successful pregnancy (2). The first *in vitro* fertilization (IVF) baby was born in 1978 (3). Since then, ART has developed rapidly, resulting in the birth of millions of babies worldwide and achieving international public acceptance. The introduction of trans vaginal oocyte aspiration (4), the use of GnRH analogs—both agonist and antagonist (5, 6), and the development of embryological laboratory facilities and technology (7) has improved the safety and success rates of ART. In 1983, the first human pregnancy achieved using embryo cryopreservation was reported (8). In 1990, preimplantation genetic diagnosis was introduced (9) and in 1992 the introduction of intracytoplasmic sperm injection (ICSI) (10). ICSI has allowed men with obstructive and some with non-obstructive azoospermia to have their own genetic children (11). Reproductive medicine in general, and ART specifically, has integrated those and other important breakthroughs into daily routine practice very quickly and further breakthroughs will open new strategies and opportunities for better preventive, accurate, and personalized reproductive medicine.

Introducing new technology into reproductive medicine always creates moral debates. This is not surprising since these technologies imply consequences not only for the gamete holders but also for their offspring. On the other hand, some of those techniques, once ready safe and efficient, might solve present ethical dilemmas such as gamete donation vs. artificial own gametes. The moral challenge that obscures other moral issues is the very large issue of care disparity that varies across the globe, with only a small proportion of infertile people currently able to access any care beyond ovarian stimulation. Infertility and treatment with ART have been identified as a field with a significant health disparity (12). Creation of a family is a basic human right. Economic, racial, ethnic, geographic, social, and cultural disparities exist in access to fertility treatments as well as in treatment outcomes. Global action and research are needed to understand disparities in treatment success and to improve treatment methods to reduce those disparities. All assisted reproductive technology (ART) stakeholders should address the existing barriers to infertility care. Clinicians should engage in efforts to develop simplified and lower-cost methods of treatment so that the cost burdens of infertility care can be reduced (13).

In this short communication I will try and bring up only some of the challenges and breakthroughs that I expect to be ready in the relatively near future. I will shortly deal with “artificial gametes,” “genetic engineering,” and non-invasive preimplantation genetic testing. These technologies may create further dramatic changes in the field of assisted reproductive technology. However, when reporting on challenges these days, one cannot ignore the great challenge that we are facing globally due to the Covid-19 pandemic. Coronavirus disease (COVID-19) is caused by a new strain of Coronavirus (SARS-CoV-2) discovered in 2019 and not previously identified in humans, and is probably now the greatest medical challenge of the world (14–16). This global challenge affects millions of people, including patients and staff dealing with ART. Available data on the exact effects of COVID-19 on fertility and pregnancy is scarce (17–27). According to recent publications, a SARSCoV-2 infection is unlikely to have long-term effects on male and female reproductive function, suggesting that the risks of ART/IVF are not altered by the COVID-19 pandemic (28–30). However, although reassuring, SARS-CoV-2 has been detected in various secretions, such as saliva, stool, urine, and the gastrointestinal tract, and thus further research is needed. At the beginning of the outbreak, major reproductive societies all over the world recommended suspension of initiation of new treatment cycles, including ovulation induction, intrauterine inseminations (IUIs), and *in vitro* fertilization (IVF) including retrievals and frozen embryo transfers, as well as non-urgent gamete cryopreservation. Furthermore, they suggested at that time to consider cancellation of all embryo transfers, whether fresh or frozen (31–35). However, with accumulating data and based on the most recent epidemiologic data on COVID-19 and pregnancy, there is no evidence to suggest increased risk for mothers or fetuses. Recent evidence suggests no association of vertical transmission and malformations, and the management of pregnant patients should be individualized based on obstetrical indications and maternal/fetal health status.

ART treatments are gradually resuming globally, with special caution and prevention actions which differ across the globe but with the aim to reduce potential hazards to the infertile couple, their potential offspring, and staff. With emergence of effective vaccines, the challenge is coping with the medical, social, and economic consequences of this crisis and its impact on societies in general and ART specifically (36).

## Artificial Gametes

One of the big challenges in ART is the use of stem cells to try and help infertile as well as same sex couples to have biological children. Although success has been achieved in mice, the use of “artificial gametes” to treat infertility is still questionable, mainly due to the fact that embryonic stem cells (ES) or induced pluripotent stem cells (iPS), while converting into precursors germ cells (PGCs), undergo global epigenetic reprogramming (37–39). Furthermore, during *in vitro* differentiation of stem cells into gametes they have to undergo meiosis which is another obstacle that makes the process even more complicated (40). Hendriks et al. (41) were able to develop a culture system in which PGC-like cells (PGCLCs) were obtained successfully via epiblast like cells (EpiLCs), starting with mice ES/iPS cells. These male PGCLCs were then transplanted into the seminiferous tubules of genetically infertile mice and contributed to sperm production. The sperm was found to be functional and resulted in fertile offspring. However, some of those iPS cell lines resulted in teratomas upon transplantation. In another study, haploid spermatid-like cells from PGCLCs co-cultured with neonatal testicular somatic cells and exposed to morphogens and sex hormones produced fertile offspring after using IVF-ICSI (42). Similarly, female PGCLCs were aggregated with gonadal somatic cells and transplanted in ovarian bursa of immuno-compromised mice, producing healthy fertile offspring, although part of the eggs had epigenetic defects (43).

To conclude, it seems that, in order to move from the bench to the clinic, a lot more needs to be accomplished. Safety issues and complex legal and ethical issues still remain when applying these technologies (44–46).

An alternative approach to overcome some of the obstacles is using very small embryonic-like stem cells (VSELs), however the very existence of VSELs is not well-accepted. The researchers that do believe in their existence assume that VSELs probably maintain life-long tissue homeostasis, serve as a backup pool for adult stem cells, and are mobilized under stress conditions. Furthermore, an imbalance in VSELs function may result in cancer (47). VSELs spontaneously differentiate *in vitro* into oocyte- and sperm-like structures (48–51). In those studies, only, the niche obtained by the somatic cells in the culture dish was necessary to induce meiosis. These findings, however, need confirmation. VSELs have been reported in chemoablated mouse ovary (52) and testis (53) and some researchers claim that transplanting mesenchymal cells (MSCs) in chemoablated mouse ovary and testis resulted in the birth of offspring (54). Preliminary results have also been obtained in women with transplantation of autologous MSCs into POF ovaries (55, 56), and the first baby was born to an idiopathic POF woman in 2016 (57).

## Genetic Engeeniring

Genetic engineering has been around for some time. However, the first birth of two twin girls was reported only as recently as 2018. This was the result of an “experiment” conducted by He Jiankui with a couple undergoing IVF in which the male was an HIV carrier. Using CRISPR technology, the CCR5 gene, which enables HIV infection, was disabled. Despite its increased precision, the risk of unexpected and undesired changes to a gene that is able to carry unpredictable consequences cannot be controlled and safety continues to be a pressing concern. Genetic engineering raises once again the issue of using technology without enough scientific evidence to support safety (58).

Furthermore, the procedure is only relevant nowadays for single gene therapy while most of the existing disorders are multigenetic. Further development of this technique in the future will probably enable dealing with up to thousands of genes at the same time and further research will make the technique reliable, efficient, and safe. Using this technology in somatic therapeutic interventions might overcome obstacles of “conventional” medical treatments (59), however, dealing with germ line interventions raise several technical and ethical issues that must be addressed. CRISPR-Cas9, and/or other gene editing technologies, might in the future evolve to be a very powerful tool to deal with different health problems.

## Non-Invasive Preimplantation Genetic Testing

Today it is possible to elucidate the entire single nucleotide-(SNV), copy number-(CNV), and structural (SV) variation of the human genome as well as comprehensive testing of the human genome by integrating massively-parallel sequencing (“next generation sequencing”) approaches together with advanced bioinformatics. These technological advances are being used to explore underlying causes of male and female infertility as well as preimplantation genetic testing (PGT). In 2016, Reigstad et al. (60) described obtaining and sequencing free DNA dripped by embryos into the culture medium, creating a new non-invasive and elegant perspective preimplantation genetic testing tool, using non-invasive chromosomal screening (NICS). Recently the results of three genetic analyzes were compared (61). NICS were compared to invasive PGT-A blastocyst biopsy in the same cultured blastocysts and the total DNA obtained from the same blastocysts were donated for research. NICS had 20% false positive results compared to 50% using PGT-A both compared with total DNA blastocyst screening as a gold standard. Several papers have recently described the births of healthy children from euploid blastocysts selected by NICS in IVF programs in couples carrying genetic alterations such as Robertsonian or balanced translocations and chromosomal inversions (62, 63). The validity of NICS in cases of repeated implantation failure, recurrent miscarriage, or advanced age is yet to be attested. NICS is a promising method that may provide another tool in our IVF toolbox to further improve our “take home healthy babies” rates.

### Maternal and Fetal Implications of Art

Assisted reproduction cycles usually involve exposure to supra physiological levels of estradiol, exogenous gonadotropins, and multiple ovarian punctures, all potentially carcinogenic. Most concern surrounds the risks of breast, endometrial, and ovarian cancers after such exposure.

Studies investigating breast cancer risks in women who underwent assisted reproduction are inconsistent. Although some studies have shown an increased breast cancer risk (64), most studies do not show an overall increase of breast cancer in exposed women (65, 66). Another study suggested an increased risk of *in situ* breast cancer (67) and another suggested a possible increased risk within subgroups of patients (68).

Most studies investigating endometrial cancer risk in exposed populations to ART have not found a significant increased risk (67), besides patients who have been exposed to unopposed estrogens for long periods.

A recent Swedish study, as well as a British study (67, 69) have suggested that women who have gone through ART have a higher risk of ovarian cancer and borderline ovarian tumors. However, they claim that at least part of the risk seems to be due to the underlying infertility and not the treatment.

Others (66), found no association between fertility drugs and ovarian cancer risk.

Due to those ongoing inconsistency cancer risk results of patients undergoing ART treatments, further large scale and long-term analysis are still needed.

An increasing number of children worldwide are born after the use of fertility treatments. However, it remains unclear whether the treatment affects the risk of childhood diseases and whether any associations observed are due to the use of specific drugs, the use of specific procedures, or the underlying infertility.

Multiple birth rates after fertility treatment are still high in many countries. Multiple births are associated with increased rates of preterm birth and low birth weight babies, in turn increasing the risk of severe morbidity for the children. Elective single-embryo transfer, particularly in combination with frozen-embryo transfer and milder stimulation in ovulation induction/intrauterine insemination, to avoid multi follicular development are effective strategies to decrease multiple birth rates while still achieving acceptable live-birth rates (67). However, ART singletons are also at increased risk of adverse obstetric and perinatal outcomes. A meta-analysis of 11 studies demonstrated that singletons born after the transfer of frozen thawed embryos had better obstetric and perinatal outcome as compared with those after the transfer of fresh IVF embryos (68).

On the contrary to the studies on adverse obstetric and perinatal outcome, in a recent retrospective cohort study looking at pediatric cancer and ART, based on a Danish population-based registry data and the Danish Infertility Cohort that included 1,085,172 children born in Denmark between 1996 and 2012 (69), they found that only the use of frozen embryo transfer, compared with children born to fertile women, was associated with a small but statistically significant increased risk of childhood cancer. In this particular study, the use of other types of fertility treatment examined was not found to be associated with increased risk of childhood cancer.

Thus, large scale, well-controlled epidemiological studies are necessary. Greater work is also necessary to identify whether the increase in obstetric, perinatal, and health impacts observed in ART children are the direct result of the ART procedure itself, or a result of the underlying subfertility of the parents. Although evidence suggests that altered DNA methylation and impaired placental development may contribute to the adverse outcomes in ART children, more studies are needed to examine whether altered epigenetic regulations are the underlying mechanism or the consequence of aberrant embryo development. As genetics and many parental characteristics cannot be altered, careful further studies to identify the optimal ART procedures that maximize both perinatal and long-term maternal and offspring health outcomes are necessary.

## Conclusions

To conclude, reproductive medicine in general and ART particularly are one of the leading dynamic developing fields in human medicine. However, many questions still remain unanswered and new concerns and challenges constantly arise. We clinicians, embryologists, and scientists dealing with our patients are very much privileged to stand on the “shoulders of our ancestors” and it is our obligation to approach new scientific outbreaks with caution and discuss the moral dilemmas of introducing those new technologies on behalf of the potential benefit to our patients while also ensuring that moral objections are not based on misunderstanding of the technique and prejudice as opposed to substantive arguments.

## Author Contributions

The author confirms being the sole contributor of this work and has approved it for publication.

## Conflict of Interest

The author declares that the research was conducted in the absence of any commercial or financial relationships that could be construed as a potential conflict of interest.

## Publisher's Note

All claims expressed in this article are solely those of the authors and do not necessarily represent those of their affiliated organizations, or those of the publisher, the editors and the reviewers. Any product that may be evaluated in this article, or claim that may be made by its manufacturer, is not guaranteed or endorsed by the publisher.
